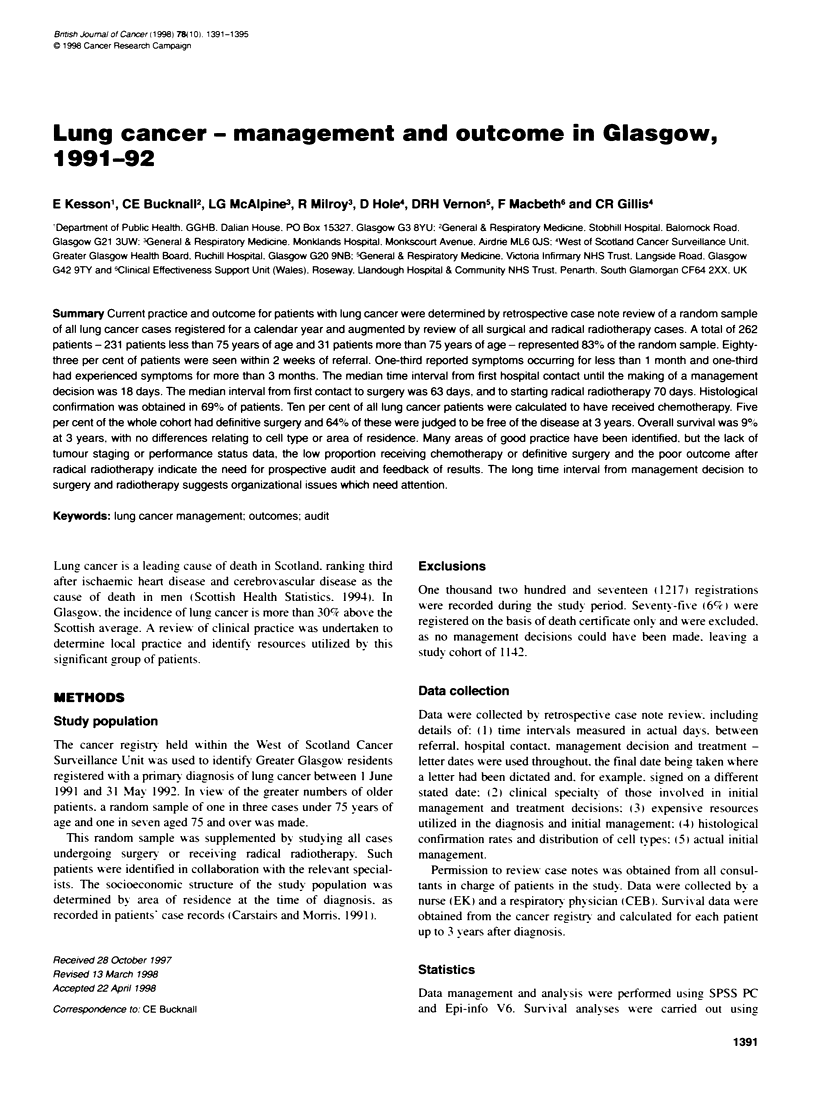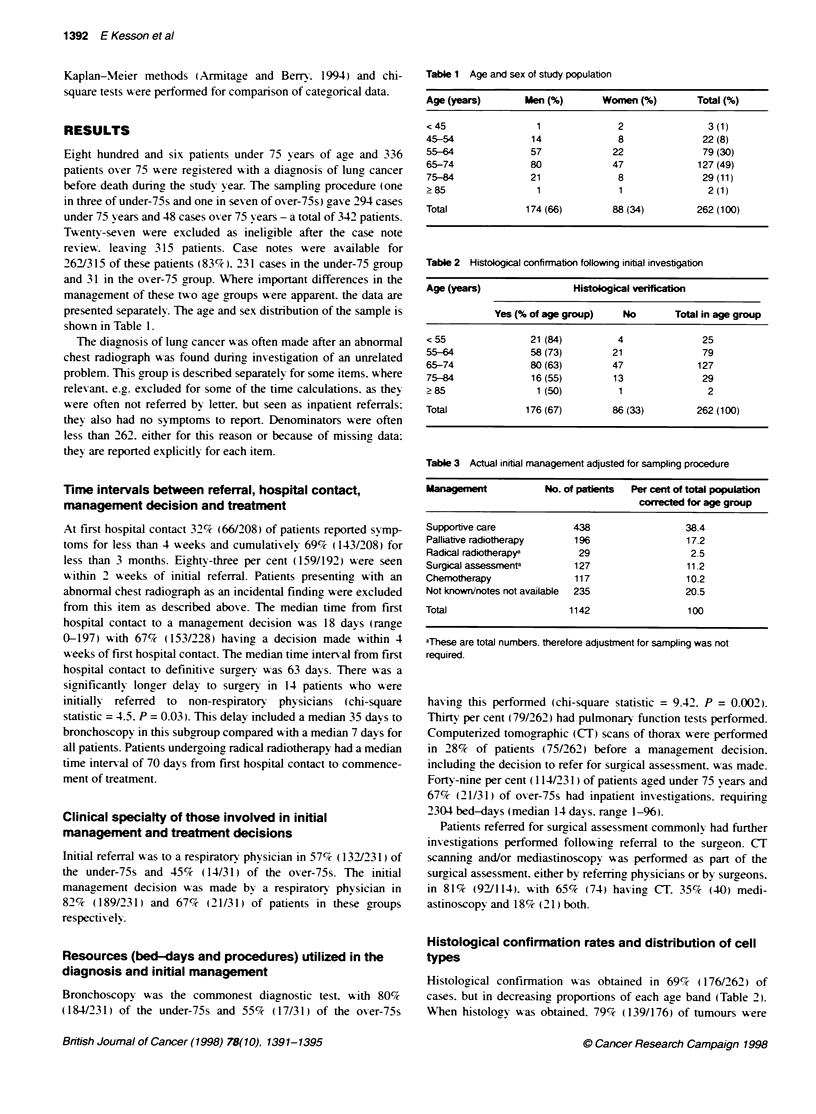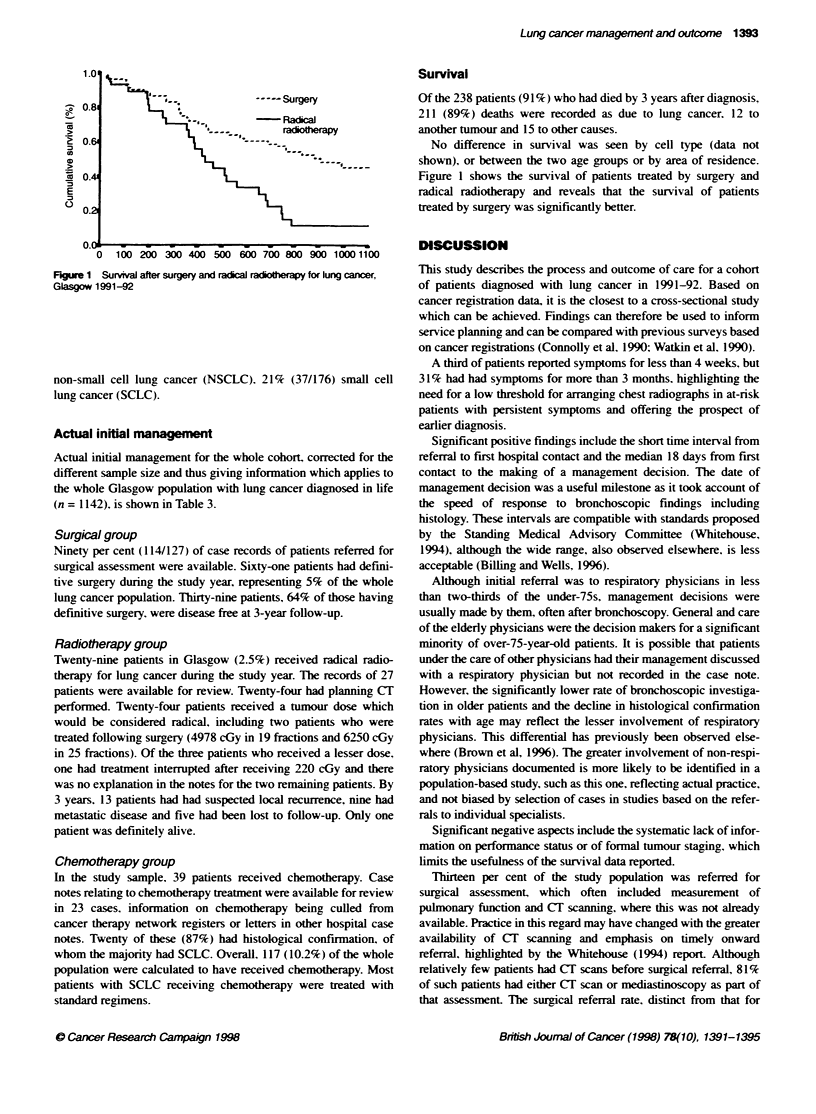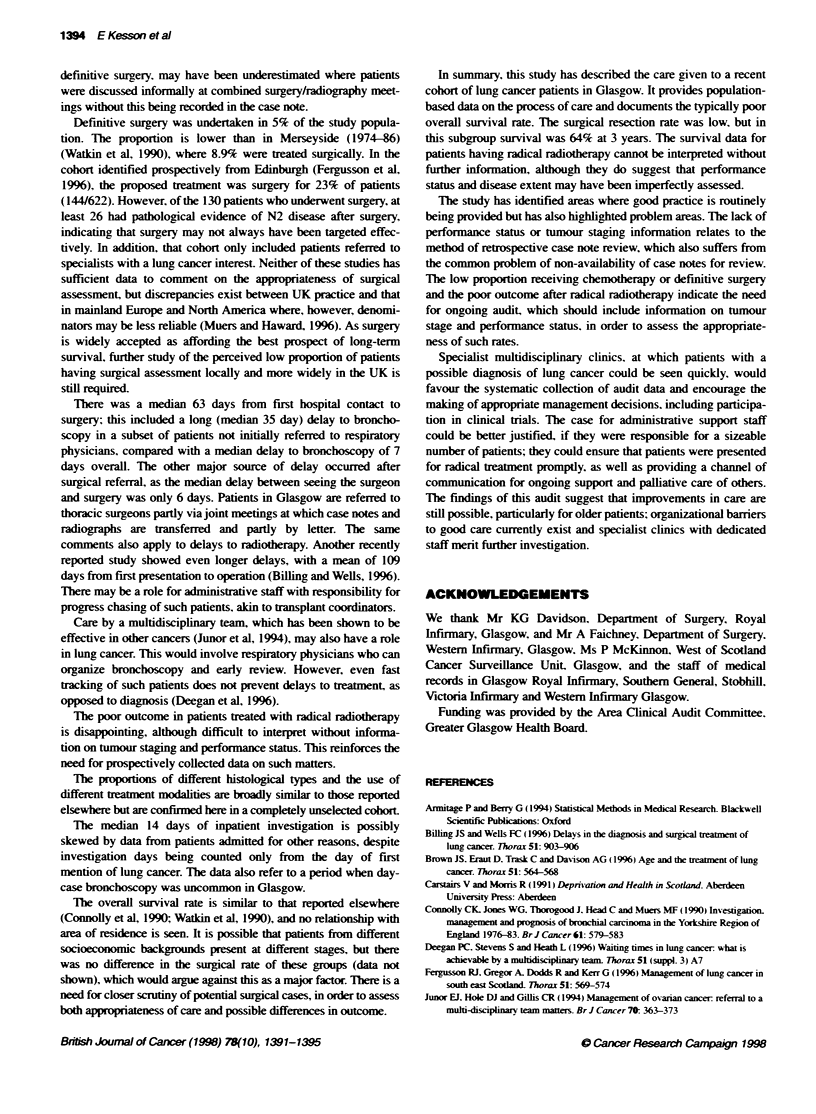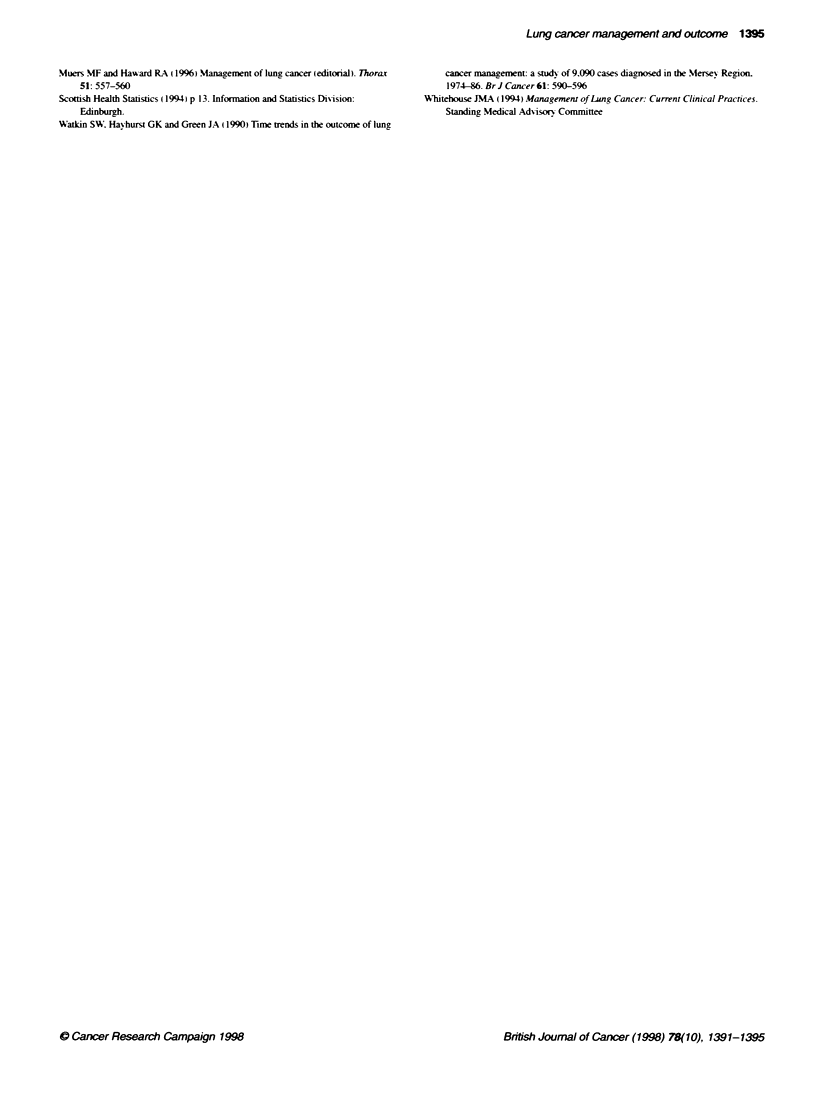# Lung cancer--management and outcome in Glasgow, 1991-92.

**DOI:** 10.1038/bjc.1998.690

**Published:** 1998-11

**Authors:** E. Kesson, C. E. Bucknall, L. G. McAlpine, R. Milroy, D. Hole, D. R. Vernon, F. Macbeth, C. R. Gillis

**Affiliations:** Department of Public Health, GGHB, Glasgow, UK.

## Abstract

Current practice and outcome for patients with lung cancer were determined by retrospective case note review of a random sample of all lung cancer cases registered for a calendar year and augmented by review of all surgical and radical radiotherapy cases. A total of 262 patients - 231 patients less than 75 years of age and 31 patients more than 75 years of age - represented 83% of the random sample. Eighty-three per cent of patients were seen within 2 weeks of referral. One-third reported symptoms occurring for less than 1 month and one-third had experienced symptoms for more than 3 months. The median time interval from first hospital contact until the making of a management decision was 18 days. The median interval from first contact to surgery was 63 days, and to starting radical radiotherapy 70 days. Histological confirmation was obtained in 69% of patients. Ten per cent of all lung cancer patients were calculated to have received chemotherapy. Five per cent of the whole cohort had definitive surgery and 64% of these were judged to be free of the disease at 3 years. Overall survival was 9% at 3 years, with no differences relating to cell type or area of residence. Many areas of good practice have been identified, but the lack of tumour staging or performance status data, the low proportion receiving chemotherapy or definitive surgery and the poor outcome after radical radiotherapy indicate the need for prospective audit and feedback of results. The long time interval from management decision to surgery and radiotherapy suggests organizational issues which need attention.


British Journal of Cancer (1998) 78(10), 1391–1395
© 1998 Cancer Research Campaign

### Lung cancer – management and outcome in Glasgow, 1991–92

E Kesson[1], CE Bucknall[2], LG McAlpine[3], R Milroy[3], D Hole[4], DRH Vernon[5], F Macbeth[6] and CR Gillis[4]

[1]Department of Public Health, GGHB, Dalian House, PO Box 15327, Glasgow G3 8YU; [2]General & Respiratory Medicine, Stobhill Hospital, Balornock Road, Glasgow G21 3UW; [3]General & Respiratory Medicine, Monklands Hospital, Monkscourt Avenue, Airdrie ML6 0JS; [4]West of Scotland Cancer Surveillance Unit, Greater Glasgow Health Board, Ruchill Hospital, Glasgow G20 9NB; [5]General & Respiratory Medicine, Victoria Infirmary NHS Trust, Langside Road, Glasgow G42 9TY and [6]Clinical Effectiveness Support Unit (Wales), Roseway, Llandough Hospital & Community NHS Trust, Penarth, South Glamorgan CF64 2XX, UK

**Summary** Current practice and outcome for patients with lung cancer were determined by retrospective case note review of a random sample of all lung cancer cases registered for a calendar year and augmented by review of all surgical and radical radiotherapy cases. A total of 262 patients – 231 patients less than 75 years of age and 31 patients more than 75 years of age – represented 83% of the random sample. Eighty-three per cent of patients were seen within 2 weeks of referral. One-third reported symptoms occurring for less than 1 month and one-third had experienced symptoms for more than 3 months. The median time interval from first hospital contact until the making of a management decision was 18 days. The median interval from first contact to surgery was 63 days, and to starting radical radiotherapy 70 days. Histological confirmation was obtained in 69% of patients. Ten per cent of all lung cancer patients were calculated to have received chemotherapy. Five per cent of the whole cohort had definitive surgery and 64% of these were judged to be free of the disease at 3 years. Overall survival was 9% at 3 years, with no differences relating to cell type or area of residence. Many areas of good practice have been identified, but the lack of tumour staging or performance status data, the low proportion receiving chemotherapy or definitive surgery and the poor outcome after radical radiotherapy indicate the need for prospective audit and feedback of results. The long time interval from management decision to surgery and radiotherapy suggests organizational issues which need attention.

**Keywords:** lung cancer management; outcomes; audit

Lung cancer is a leading cause of death in Scotland, ranking third after ischaemic heart disease and cerebrovascular disease as the cause of death in men (Scottish Health Statistics, 1994). In Glasgow, the incidence of lung cancer is more than 30% above the Scottish average. A review of clinical practice was undertaken to determine local practice and identify resources utilized by this significant group of patients.

### METHODS

### Study population

The cancer registry held within the West of Scotland Cancer Surveillance Unit was used to identify Greater Glasgow residents registered with a primary diagnosis of lung cancer between 1 June 1991 and 31 May 1992. In view of the greater numbers of older patients, a random sample of one in three cases under 75 years of age and one in seven aged 75 and over was made.

This random sample was supplemented by studying all cases undergoing surgery or receiving radical radiotherapy. Such patients were identified in collaboration with the relevant specialists. The socioeconomic structure of the study population was determined by area of residence at the time of diagnosis, as recorded in patients' case records (Carstairs and Morris, 1991).

*Received 28 October 1997*
*Revised 13 March 1998*
*Accepted 22 April 1998*

*Correspondence to:* CE Bucknall

### Exclusions

One thousand two hundred and seventeen (1217) registrations were recorded during the study period. Seventy-five (6%) were registered on the basis of death certificate only and were excluded, as no management decisions could have been made, leaving a study cohort of 1142.

### Data collection

Data were collected by retrospective case note review, including details of: (1) time intervals measured in actual days, between referral, hospital contact, management decision and treatment – letter dates were used throughout, the final date being taken where a letter had been dictated and, for example, signed on a different stated date; (2) clinical specialty of those involved in initial management and treatment decisions; (3) expensive resources utilized in the diagnosis and initial management; (4) histological confirmation rates and distribution of cell types; (5) actual initial management.

Permission to review case notes was obtained from all consultants in charge of patients in the study. Data were collected by a nurse (EK) and a respiratory physician (CEB). Survival data were obtained from the cancer registry and calculated for each patient up to 3 years after diagnosis.

### Statistics

Data management and analysis were performed using SPSS PC and Epi-info V6. Survival analyses were carried out using

Kaplan–Meier methods (Armitage and Berry, 1994) and chi-square tests were performed for comparison of categorical data.

### RESULTS

Eight hundred and six patients under 75 years of age and 336 patients over 75 were registered with a diagnosis of lung cancer before death during the study year. The sampling procedure (one in three of under-75s and one in seven of over-75s) gave 294 cases under 75 years and 48 cases over 75 years – a total of 342 patients. Twenty-seven were excluded as ineligible after the case note review, leaving 315 patients. Case notes were available for 262/315 of these patients (83%), 231 cases in the under-75 group and 31 in the over-75 group. Where important differences in the management of these two age groups were apparent, the data are presented separately. The age and sex distribution of the sample is shown in Table 1.

The diagnosis of lung cancer was often made after an abnormal chest radiograph was found during investigation of an unrelated problem. This group is described separately for some items, where relevant, e.g. excluded for some of the time calculations, as they were often not referred by letter, but seen as inpatient referrals; they also had no symptoms to report. Denominators were often less than 262, either for this reason or because of missing data; they are reported explicitly for each item.

### Time intervals between referral, hospital contact, management decision and treatment

At first hospital contact 32% (66/208) of patients reported symptoms for less than 4 weeks and cumulatively 69% (143/208) for less than 3 months. Eighty-three per cent (159/192) were seen within 2 weeks of initial referral. Patients presenting with an abnormal chest radiograph as an incidental finding were excluded from this item as described above. The median time from first hospital contact to a management decision was 18 days (range 0–197) with 67% (153/228) having a decision made within 4 weeks of first hospital contact. The median time interval from first hospital contact to definitive surgery was 63 days. There was a significantly longer delay to surgery in 14 patients who were initially referred to non-respiratory physicians (chi-square statistic = 4.5, $P = 0.03$). This delay included a median 35 days to bronchoscopy in this subgroup compared with a median 7 days for all patients. Patients undergoing radical radiotherapy had a median time interval of 70 days from first hospital contact to commencement of treatment.

### Clinical specialty of those involved in initial management and treatment decisions

Initial referral was to a respiratory physician in 57% (132/231) of the under-75s and 45% (14/31) of the over-75s. The initial management decision was made by a respiratory physician in 82% (189/231) and 67% (21/31) of patients in these groups respectively.

### Resources (bed–days and procedures) utilized in the diagnosis and initial management

Bronchoscopy was the commonest diagnostic test, with 80% (184/231) of the under-75s and 55% (17/31) of the over-75s having this performed (chi-square statistic = 9.42, $P = 0.002$). Thirty per cent (79/262) had pulmonary function tests performed. Computerized tomographic (CT) scans of thorax were performed in 28% of patients (75/262) before a management decision, including the decision to refer for surgical assessment, was made. Forty-nine per cent (114/231) of patients aged under 75 years and 67% (21/31) of over-75s had inpatient investigations, requiring 2304 bed–days (median 14 days, range 1–96).

Patients referred for surgical assessment commonly had further investigations performed following referral to the surgeon. CT scanning and/or mediastinoscopy was performed as part of the surgical assessment, either by referring physicians or by surgeons, in 81% (92/114), with 65% (74) having CT, 35% (40) mediastinoscopy and 18% (21) both.

**Table 1** Age and sex of study population

| Age (years) | Men (%) | Women (%) | Total (%) |
|---|---|---|---|
| < 45 | 1 | 2 | 3 (1) |
| 45–54 | 14 | 8 | 22 (8) |
| 55–64 | 57 | 22 | 79 (30) |
| 65–74 | 80 | 47 | 127 (49) |
| 75–84 | 21 | 8 | 29 (11) |
| ≥ 85 | 1 | 1 | 2 (1) |
| Total | 174 (66) | 88 (34) | 262 (100) |

**Table 2** Histological confirmation following initial investigation

| Age (years) | Histological verification | | |
|---|---|---|---|
| | Yes (% of age group) | No | Total in age group |
| < 55 | 21 (84) | 4 | 25 |
| 55–64 | 58 (73) | 21 | 79 |
| 65–74 | 80 (63) | 47 | 127 |
| 75–84 | 16 (55) | 13 | 29 |
| ≥ 85 | 1 (50) | 1 | 2 |
| Total | 176 (67) | 86 (33) | 262 (100) |

**Table 3** Actual initial management adjusted for sampling procedure

| Management | No. of patients | Per cent of total population corrected for age group |
|---|---|---|
| Supportive care | 438 | 38.4 |
| Palliative radiotherapy | 196 | 17.2 |
| Radical radiotherapy[a] | 29 | 2.5 |
| Surgical assessment[a] | 127 | 11.2 |
| Chemotherapy | 117 | 10.2 |
| Not known/notes not available | 235 | 20.5 |
| Total | 1142 | 100 |

[a]These are total numbers, therefore adjustment for sampling was not required.

### Histological confirmation rates and distribution of cell types

Histological confirmation was obtained in 69% (176/262) of cases, but in decreasing proportions of each age band (Table 2). When histology was obtained, 79% (139/176) of tumours were

© Cancer Research Campaign 1998

**Figure 1** Survival after surgery and radical radiotherapy for lung cancer, Glasgow 1991–92

non-small cell lung cancer (NSCLC), 21% (37/176) small cell lung cancer (SCLC).

### Actual initial management

Actual initial management for the whole cohort, corrected for the different sample size and thus giving information which applies to the whole Glasgow population with lung cancer diagnosed in life ($n$ = 1142), is shown in Table 3.

#### *Surgical group*

Ninety per cent (114/127) of case records of patients referred for surgical assessment were available. Sixty-one patients had definitive surgery during the study year, representing 5% of the whole lung cancer population. Thirty-nine patients, 64% of those having definitive surgery, were disease free at 3-year follow-up.

#### *Radiotherapy group*

Twenty-nine patients in Glasgow (2.5%) received radical radiotherapy for lung cancer during the study year. The records of 27 patients were available for review. Twenty-four had planning CT performed. Twenty-four patients received a tumour dose which would be considered radical, including two patients who were treated following surgery (4978 cGy in 19 fractions and 6250 cGy in 25 fractions). Of the three patients who received a lesser dose, one had treatment interrupted after receiving 220 cGy and there was no explanation in the notes for the two remaining patients. By 3 years, 13 patients had had suspected local recurrence, nine had metastatic disease and five had been lost to follow-up. Only one patient was definitely alive.

#### *Chemotherapy group*

In the study sample, 39 patients received chemotherapy. Case notes relating to chemotherapy treatment were available for review in 23 cases, information on chemotherapy being culled from cancer therapy network registers or letters in other hospital case notes. Twenty of these (87%) had histological confirmation, of whom the majority had SCLC. Overall, 117 (10.2%) of the whole population were calculated to have received chemotherapy. Most patients with SCLC receiving chemotherapy were treated with standard regimens.

### Survival

Of the 238 patients (91%) who had died by 3 years after diagnosis, 211 (89%) deaths were recorded as due to lung cancer, 12 to another tumour and 15 to other causes.

No difference in survival was seen by cell type (data not shown), or between the two age groups or by area of residence. Figure 1 shows the survival of patients treated by surgery and radical radiotherapy and reveals that the survival of patients treated by surgery was significantly better.

### DISCUSSION

This study describes the process and outcome of care for a cohort of patients diagnosed with lung cancer in 1991–92. Based on cancer registration data, it is the closest to a cross-sectional study which can be achieved. Findings can therefore be used to inform service planning and can be compared with previous surveys based on cancer registrations (Connolly et al, 1990; Watkin et al, 1990).

A third of patients reported symptoms for less than 4 weeks, but 31% had had symptoms for more than 3 months, highlighting the need for a low threshold for arranging chest radiographs in at-risk patients with persistent symptoms and offering the prospect of earlier diagnosis.

Significant positive findings include the short time interval from referral to first hospital contact and the median 18 days from first contact to the making of a management decision. The date of management decision was a useful milestone as it took account of the speed of response to bronchoscopic findings including histology. These intervals are compatible with standards proposed by the Standing Medical Advisory Committee (Whitehouse, 1994), although the wide range, also observed elsewhere, is less acceptable (Billing and Wells, 1996).

Although initial referral was to respiratory physicians in less than two-thirds of the under-75s, management decisions were usually made by them, often after bronchoscopy. General and care of the elderly physicians were the decision makers for a significant minority of over-75-year-old patients. It is possible that patients under the care of other physicians had their management discussed with a respiratory physician but not recorded in the case note. However, the significantly lower rate of bronchoscopic investigation in older patients and the decline in histological confirmation rates with age may reflect the lesser involvement of respiratory physicians. This differential has previously been observed elsewhere (Brown et al, 1996). The greater involvement of non-respiratory physicians documented is more likely to be identified in a population-based study, such as this one, reflecting actual practice, and not biased by selection of cases in studies based on the referrals to individual specialists.

Significant negative aspects include the systematic lack of information on performance status or of formal tumour staging, which limits the usefulness of the survival data reported.

Thirteen per cent of the study population was referred for surgical assessment, which often included measurement of pulmonary function and CT scanning, where this was not already available. Practice in this regard may have changed with the greater availability of CT scanning and emphasis on timely onward referral, highlighted by the Whitehouse (1994) report. Although relatively few patients had CT scans before surgical referral, 81% of such patients had either CT scan or mediastinoscopy as part of that assessment. The surgical referral rate, distinct from that for definitive surgery, may have been underestimated where patients were discussed informally at combined surgery/radiography meetings without this being recorded in the case note.

Definitive surgery was undertaken in 5% of the study population. The proportion is lower than in Merseyside (1974–86) (Watkin et al, 1990), where 8.9% were treated surgically. In the cohort identified prospectively from Edinburgh (Fergusson et al, 1996), the proposed treatment was surgery for 23% of patients (144/622). However, of the 130 patients who underwent surgery, at least 26 had pathological evidence of N2 disease after surgery, indicating that surgery may not always have been targeted effectively. In addition, that cohort only included patients referred to specialists with a lung cancer interest. Neither of these studies has sufficient data to comment on the appropriateness of surgical assessment, but discrepancies exist between UK practice and that in mainland Europe and North America where, however, denominators may be less reliable (Muers and Haward, 1996). As surgery is widely accepted as affording the best prospect of long-term survival, further study of the perceived low proportion of patients having surgical assessment locally and more widely in the UK is still required.

There was a median 63 days from first hospital contact to surgery; this included a long (median 35 day) delay to bronchoscopy in a subset of patients not initially referred to respiratory physicians, compared with a median delay to bronchoscopy of 7 days overall. The other major source of delay occurred after surgical referral, as the median delay between seeing the surgeon and surgery was only 6 days. Patients in Glasgow are referred to thoracic surgeons partly via joint meetings at which case notes and radiographs are transferred and partly by letter. The same comments also apply to delays to radiotherapy. Another recently reported study showed even longer delays, with a mean of 109 days from first presentation to operation (Billing and Wells, 1996). There may be a role for administrative staff with responsibility for progress chasing of such patients, akin to transplant coordinators.

Care by a multidisciplinary team, which has been shown to be effective in other cancers (Junor et al, 1994), may also have a role in lung cancer. This would involve respiratory physicians who can organize bronchoscopy and early review. However, even fast tracking of such patients does not prevent delays to treatment, as opposed to diagnosis (Deegan et al, 1996).

The poor outcome in patients treated with radical radiotherapy is disappointing, although difficult to interpret without information on tumour staging and performance status. This reinforces the need for prospectively collected data on such matters.

The proportions of different histological types and the use of different treatment modalities are broadly similar to those reported elsewhere but are confirmed here in a completely unselected cohort.

The median 14 days of inpatient investigation is possibly skewed by data from patients admitted for other reasons, despite investigation days being counted only from the day of first mention of lung cancer. The data also refer to a period when day-case bronchoscopy was uncommon in Glasgow.

The overall survival rate is similar to that reported elsewhere (Connolly et al, 1990; Watkin et al, 1990), and no relationship with area of residence is seen. It is possible that patients from different socioeconomic backgrounds present at different stages, but there was no difference in the surgical rate of these groups (data not shown), which would argue against this as a major factor. There is a need for closer scrutiny of potential surgical cases, in order to assess both appropriateness of care and possible differences in outcome.

In summary, this study has described the care given to a recent cohort of lung cancer patients in Glasgow. It provides population-based data on the process of care and documents the typically poor overall survival rate. The surgical resection rate was low, but in this subgroup survival was 64% at 3 years. The survival data for patients having radical radiotherapy cannot be interpreted without further information, although they do suggest that performance status and disease extent may have been imperfectly assessed.

The study has identified areas where good practice is routinely being provided but has also highlighted problem areas. The lack of performance status or tumour staging information relates to the method of retrospective case note review, which also suffers from the common problem of non-availability of case notes for review. The low proportion receiving chemotherapy or definitive surgery and the poor outcome after radical radiotherapy indicate the need for ongoing audit, which should include information on tumour stage and performance status, in order to assess the appropriateness of such rates.

Specialist multidisciplinary clinics, at which patients with a possible diagnosis of lung cancer could be seen quickly, would favour the systematic collection of audit data and encourage the making of appropriate management decisions, including participation in clinical trials. The case for administrative support staff could be better justified, if they were responsible for a sizeable number of patients; they could ensure that patients were presented for radical treatment promptly, as well as providing a channel of communication for ongoing support and palliative care of others. The findings of this audit suggest that improvements in care are still possible, particularly for older patients; organizational barriers to good care currently exist and specialist clinics with dedicated staff merit further investigation.

### ACKNOWLEDGEMENTS

We thank Mr KG Davidson, Department of Surgery, Royal Infirmary, Glasgow, and Mr A Faichney, Department of Surgery, Western Infirmary, Glasgow, Ms P McKinnon, West of Scotland Cancer Surveillance Unit, Glasgow, and the staff of medical records in Glasgow Royal Infirmary, Southern General, Stobhill, Victoria Infirmary and Western Infirmary Glasgow.

Funding was provided by the Area Clinical Audit Committee, Greater Glasgow Health Board.

### REFERENCES

Armitage P and Berry G (1994) Statistical Methods in Medical Research. Blackwell Scientific Publications: Oxford

Billing JS and Wells FC (1996) Delays in the diagnosis and surgical treatment of lung cancer. *Thorax* 51: 903–906

Brown JS, Eraut D, Trask C and Davison AG (1996) Age and the treatment of lung cancer. *Thorax* 51: 564–568

Carstairs V and Morris R (1991) *Deprivation and Health in Scotland*. Aberdeen University Press: Aberdeen

Connolly CK, Jones WG, Thorogood J, Head C and Muers MF (1990) Investigation, management and prognosis of bronchial carcinoma in the Yorkshire Region of England 1976–83. *Br J Cancer* 61: 579–583

Deegan PC, Stevens S and Heath L (1996) Waiting times in lung cancer: what is achievable by a multidisciplinary team. *Thorax* 51 (suppl. 3) A7

Fergusson RJ, Gregor A, Dodds R and Kerr G (1996) Management of lung cancer in south east Scotland. *Thorax* 51: 569–574

Junor EJ, Hole DJ and Gillis CR (1994) Management of ovarian cancer: referral to a multi-disciplinary team matters. *Br J Cancer* 70: 363–373

 © Cancer Research Campaign 1998

Muers MF and Haward RA (1996) Management of lung cancer (editorial). *Thorax* **51**: 557–560
Scottish Health Statistics (1994) p 13. Information and Statistics Division: Edinburgh.
Watkin SW, Hayhurst GK and Green JA (1990) Time trends in the outcome of lung cancer management: a study of 9.090 cases diagnosed in the Mersey Region. 1974–86. *Br J Cancer* **61**: 590–596
Whitehouse JMA (1994) *Management of Lung Cancer: Current Clinical Practices*. Standing Medical Advisory Committee

© Cancer Research Campaign 1998